# The Relationship between Runs of Homozygosity and Inbreeding in Jersey Cattle under Selection

**DOI:** 10.1371/journal.pone.0129967

**Published:** 2015-07-08

**Authors:** Eui-Soo Kim, Tad S. Sonstegard, Curtis P. Van Tassell, George Wiggans, Max F. Rothschild

**Affiliations:** 1 Animal Genomics & Improvement Laboratory, Beltsville Agricultural Research Center, Agricultural Research Service, United States Department of Agriculture, Beltsville, Maryland, United States of America; 2 Department of Animal Science, Iowa State University, Ames, Iowa, United States of America; University of Sydney, AUSTRALIA

## Abstract

Inbreeding is often an inevitable outcome of strong directional artificial selection but on average it reduces population fitness with increased frequency of recessive deleterious alleles. Runs of homozygosity (ROH) representing genomic autozygosity that occur from mating between selected and genomically related individuals may be able to reveal the regions affecting fitness. To examine the influence of genomic autozygosity on fitness, we used a genome-wide association test to evaluate potential negative correlations between ROH and daughter pregnancy rate (DPR) or somatic cell score (SCS) in US Jersey cattle. In addition, relationships between changes of local ROH and inbreeding coefficients (*F*) were assessed to locate genomic regions with increased inbreeding. Despite finding some decreases in fertility associated with incremental increases in *F*, most emerging local ROH were not significantly associated with DPR or SCS. Furthermore, the analyses of ROH could be approximated with the most frequent haplotype(s), including the associations of ROH and *F* or traits. The analysis of the most frequent haplotype revealed that associations of ROH and fertility could be accounted for by the additive genetic effect on the trait. Thus, we suggest that a change of autozygosity is more likely to demonstrate footprints of selected haplotypes for production rather than highlight the possible increased local autozygosity of a recessive detrimental allele resulting from the mating between closely related animals in Jersey cattle.

## Introduction

Inbreeding increases autozygosity of loci throughout the genome, some of which causes homozygosity of recessive alleles that may cause expression of an unfavorable phenotype. Although the exact number of deleterious mutations have not been estimated, it is important to note that most animals are assumed to be carriers of at least one recessively inherited disorder [1]. Previous studies have reported that individual humans would have 40–110 potentially deleterious variants and 0.4 recessive lethal mutations [2]. The frequency of a recessive lethal polymorphism in protein coding genes in some experimental species such as *Drosophila melanogaster* is expected to be substantial (25%) [3]. While autozygosity may reduce recessive deleterious allele frequency by chance, selection is the principal force that inhibits detrimental alleles from increasing in frequency [4].

Although inbreeding on average increases the expression of recessive deleterious alleles corresponding to genetic disease [5], creative use of mating systems between related individuals can increase selection response [6]. To estimate the extent of inbreeding depression, marker heterozygosity considering all fitness-influencing loci across the genome were estimated and the power to detect heterozygosity-fitness associations depended on the number of markers when only a few markers were genotyped [7]. Positive correlations between a trait of interest like reproductive fitness and level of marker heterozygosity are recognized as suggestive evidence of inbreeding depression [8]. Heterozygosity across a few microsatellite loci has been used to infer an inbreeding coefficient (*F*), but in an outbred natural population correlations between *F* and sparse molecular markers were relatively weak [9]. Recent development of high density SNP genotyping tools allows more precise examination of inbreeding even without records. Indeed, the genomic inbreeding coefficient estimated using continuous homozygous SNPs known as runs of homozygosity (ROH) was found to correlate well with the pedigree inbreeding coefficient estimated from a relatively small human cohort [10]. Varying lengths of ROH provide generational information about inbreeding levels in a reference population [11]. Moreover, the presence of unevenly distributed ROH across the genomes of individuals in European human populations suggested regional selection for adaptive variants [12]. Europeans generally have ROH shorter than 1.5 Mb, implying ancient linkage disequilibrium (LD) patterns or the inheritance of common haplotypes from both parents, whereas larger ROH reflect recent parental relatedness or a genomic region conferring a selective advantage [10]. In cattle, ROH analysis of two Holstein cow populations under 40 years of intense or no selection for milk production revealed most ROH ranged between 1.5 and 5.0 Mb across both populations, while ROH lengths of 10 Mb or higher could be found in the population under selection [13]. Because genomic autozygosity based on ROH allows for potential differentiation of inbreeding from selection for a quantitative trait [14], genomic regions encompassing extensive ROH potentially contain gene variants associated with genetic improvement in livestock [15].

The combination of intense selection for improved milk production based and artificial insemination from a limited number of related elite sires has reduced effective population size in most dairy breeds, and possibly also reduced average animal fitness. For example, a negative genetic correlation between reproduction and milk yield is currently one of the obstacles for accelerated genetic improvement of fertility [16]. While most studies emphasize the overall effect of inbreeding levels on reproduction [17], few studies report the correlation between local genomic autozygosity and phenotypes commonly associated with inbreeding depression. For the purpose of identifying the influence of recent inbreeding on two fitness traits, an association test of phenotypes and ROH-based autozygosity, representing a proxy for potential depression due to inbreeding, was performed using BovineSNP50 genotypes from 1,602 U.S. Jerseys. In addition, association between ROH and *F* was examined to identify if specific genomic regions contribute more frequently to overall autozygosity. These ROH were then compared to examine if there was a genetic effect on two fitness traits. Furthermore, the whole genome was scanned using haplotype windows allowing comparison between the most frequent haplotype alleles relative to ROH. These latter analyses provided insights of inbreeding depression relative to locus-specific (local hypothesis) and general genome-wide effects (general hypothesis), in an attempt to elucidate possible mechanisms underlying associations between genetic diversity and fitness [18].

## Materials and Methods

### Animals and genotypes

Pedigree information obtained from the USDA-Animal Improvement Programs Laboratory (Beltsville, MD) on 19,966 registered Jersey animals born in the United States between 1953 and 2008 was used to compute inbreeding coefficients (*F*) [19] using Pedigree Viewer [20]. Additionally, recorded phenotypes including production ability, fertility and disease related traits were collected. Inbreeding coefficients for animals with no parents or ancestors in the pedigree records were set to zero. Modern dairy cattle populations are comprised of both inbred (*F* ~ 0.1) and outbred structure because of intensive use of a small number of influential males selected for artificial insemination (AI) and mated to cows that probably originated from common ancestors born more than three generations ago, which creates complex pedigree structures consisting of multiple inbreeding loops. The genotyped animals consisted of 1,219 male and 383 female Jersey animals that were born between the 1960s and the 2000s. Most genotyped animals (N = 1,280) were born after 1990s, but frozen semen samples maintained for AI enabled us to genotype influential ancestors that were born before 1990s (N = 322), which may affect genetic polymorphisms of contemporary Jersey cattle. To reduce population stratification, animals were only sampled from small half-sib families (n = 3–30) without considering relationships in the extended pedigree. Then, the Illumina BovineSNP50 beadchip (Illumina, CA) was used to genotype 1,602 animals sampled from the U.S. Jersey cattle described above. The PLINK software [21] was used to screen SNPs based on minor allele frequency (MAF>0.01), Hardy-Weinberg Equilibrium (HWE) test (-log_10_p<3), genotyping rate (>0.8), and individuals with missing genotypes (<20%) and data were removed that did not conform. Finally, 36,869 SNPs were selected in autosomal chromosomes and used in all subsequent analyses. SNP genome coordinates were obtained from the bovine genome reference assembly UMD 3.1.

### Definition of autozygous genomic region

Two approaches were used to define a homozygous genomic region. First, runs of homozygosity (ROH) were determined under the following criteria using a Perl script according to the modified methods that were suggested for the analysis of data in humans [10]. Considering that the cattle genome has in general regions with low density of SNPs, the criteria for defining genomic regions as ROH was 50 or more consecutive homozygous SNPs which is equivalent to approximately a 2 Mb region, and which allowed detection of homozygous regions encompassing 2–5 Mb region that are likely to originate from common ancestors for up to 10–25 generations ago [22]. For the purpose of defining locus autozygosity, we calculated the sum of ROH status (0 or 1) at each SNP across all genotyped animals. Furthermore, the summation of ROH across all marker genotypes for an individual was considered as overall genomic autozygosity (FROH), and this calculation of genomic inbreeding was then compared to the inbreeding coefficient (*F*) estimated based on pedigree (FPED). Haplotypes were obtained using fastphase [23] and the analyses of ROH were performed using Perl and R scripts.

### Population structure

Principal components analysis (PCA) was performed using genotypes and ROH separately to examine and correct the potential population structure [24]. Association of *F* and ROH was assessed with or without adjusting for potential stratification. Therefore, the homozygous state of SNPs defined by ROH was used to conduct the analysis of principal components. Principal components (PCs) are obtained, which are included as covariates in a multiple regression model involving ROH and the inbreeding coefficient, which enables to adjust for underlying population structure [24]. To calculate principal components, the adegenet package [25] was used.

### Associations between ROH and inbreeding coefficient

To evaluate associations between ROH at individual loci and inbreeding coefficients, a linear model was used without transforming FPED. The regression model was *y* = *β*_0_ + *β*_1_ *H* + *e* where, *y* was the inbreeding coefficient (FPED) of an individual, *β*_*0*_ was the intercept of the equation, *β*_*1*_ was the coefficient of the predictor, *H* is the ROH as a particular locus (0 or 1), and *e* is the random error. To correct the effect of population stratification, principal components (PC_n_) from PCA were included as covariates in the linear model [24], $y=\beta_{0}+\beta_{1}H+\sum_{i=1}^{n}PC_{i}+e$, where *PCi* is *i*th principal component obtained using ROH and n is the number of PCs. Additionally, birth year of an animal was analyzed as a response (*y*) using the same model to assess the change of homozygosity. Statistical thresholds for a genome wide search of associations of genomic homozygosity and pedigree based inbreeding coefficient were determined empirically using permutation tests [26]. Inbreeding coefficient (FPED) was permuted repeatedly to obtain thresholds using the most significant result of each permutation was reserved [27]. The genome-wide critical values (1% = significant level and 5% = suggestive level) were obtained by 1,000 permutations of association tests between FPED and ROH in the whole genome. R and Perl scripts were used to detect associations and to calculate thresholds.

### Association of ROH and haplotype homozygosity with phenotypes

Association of ROH and a trait was examined across the genome. In addition, the sliding window approach partitioned the haplotype homozygosity (HH) effect of each allele contributing to overall homozygosity. The predicted transfer ability (PTA) of daughter pregnancy rate and somatic cell count (SCS) in milk records of U.S. Jersey, which are genetic values after adjusting for environmental and polygenic effects, were obtained from USDA-AIPL (https://aipl.arsusda.gov/). Daughter pregnancy rate (DPR) was calculated from days open and directly relates to the proportion of females eligible to become pregnant in a 21 day period [28]. The association between haplotype or ROH and each trait was evaluated using linear regression [26], *y* = *β*_0_ + *β*_1_ *G* + *e*, where *y* is PTA of DPR or SCS of an individual, *β*_*0*_ is the intercept, and *β*_*1*_ is a vector of recessive genetic effect. *G* is an indicator variable for the effect of ROH of an individual, and *e* is the random error. In this analysis, the effect of population structure in PTA was reduced by statistical adjustment using a linear mixed model including the effect of genetic relationships and environmental factors. The genome-wide statistical significance level was determined using 1,000 permutation tests [27] as described above.

Since ROH reflects the sum of haplotype homozygosity, the additive effect is inestimable. Therefore, associations of the most frequent haplotype and fertility (DPR) were also estimated using the additive or recessive model for the further understanding of genetic effect of the haplotype that is highly correlated with ROH. To estimate the additive genetic effect of the most common haplotype, the number of alleles of the most frequent haplotype are considered SNP-like genotypes (*G* = 0, 1 or 2) using the same model for ROH-trait association. Similarly, the homozygote of the most frequent haplotype was assumed to encompass the recessive allele and other haplotypes were assumed to have no recessive allele. The genetic effect was evaluated using the same model used for association test of ROH and trait by replacement of homozygote status indicator with an allele number in the sliding window. Finally, functional annotation for genes located in the regions identified from the association test between ROH and phenotypes was performed using the Enrichr software [29].

## Results

### ROH and inbreeding

The length of an autozygous haplotype originating from a common ancestor 10 generations in the past was expected to be approximately 5 Mb, which is close to a typical size of ROH using thresholds ranging from 30–50 homozygous SNPs (S1 Table). At a 50 SNP threshold, the mean and median of homozygous fragments were 8.48 and 6.09 Mb, respectively. Correlations of FPED and FROH were 0.6–0.7 under the various definitions of ROH (S1 Table), while the level of ROH was independent of the definition of ROH. In all cases, FROH was higher than the mean FPED, implying that pedigree based inbreeding coefficients could be underestimated. Using pedigrees, we found that almost all individuals shared at least one common ancestor within 3 or 4 generations except founders and their offspring. In some individuals, particularly contemporary animals, the ROH extended over 10 Mb, suggesting derivation from recent common ancestors only 5 generations ago. We could not confirm due to limited resources of DNA and the presence of complex inbreeding loops, as to whether a ROH shared an identical allele from a common ancestor. In the genotyped animals with an assumption of no genetic relationships among founder animals, inbreeding coefficients ranged from 0.02 to 0.29 and the mean and median *F* were estimated to be 0.061 and 0.058, respectively. The inbreeding coefficient based on pedigree was nearly approximated by the estimated genomic autozygosity based on the sum of ROH per animal genome (S1 Fig).

**Fig 1:**
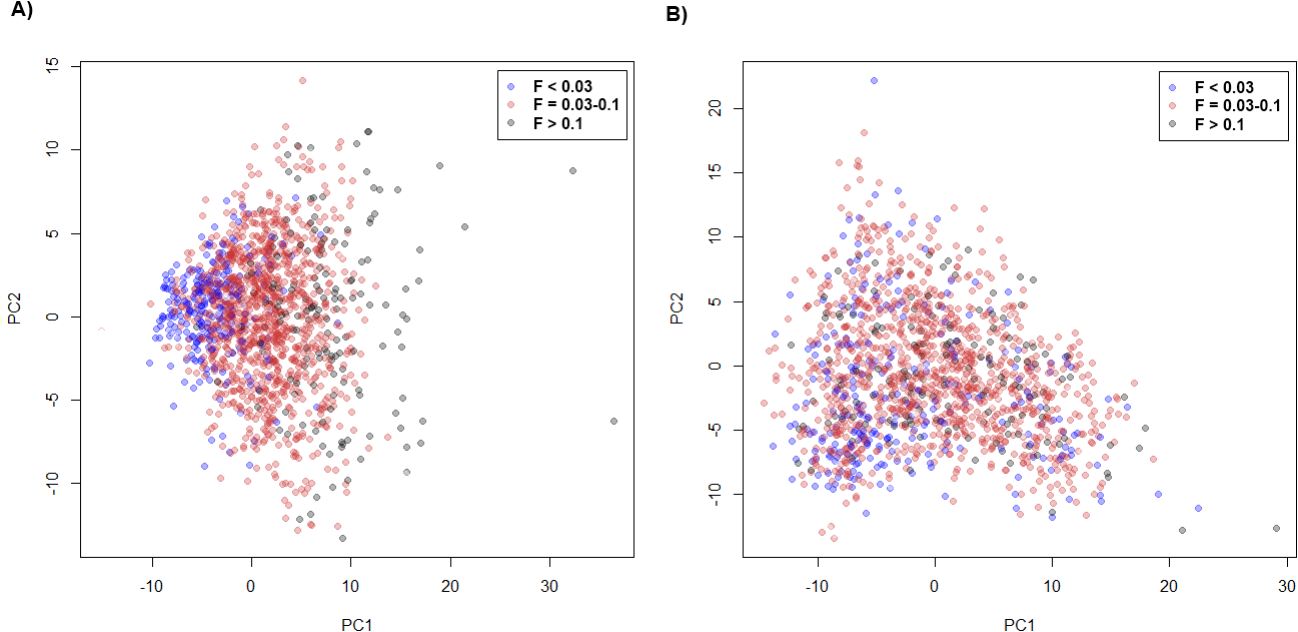
Principal component analysis of Jersey cattle using ROH (A) and SNP genotypes (B). Principal component 1 (PC1, x axis) and principal component 2 (PC2, y axis) are plotted. Three groups that are classified based on inbreeding coefficient (FPED) are indicated with three different colors. Blue, red, and black circles represent individuals with FPED<0.03, FPED = 0.03–0.10, and FPED>0.10, respectively.

Although obvious sub-populations were not separated, ROH based PCA showed that the levels of inbreeding coefficients could be roughly separated by PC1 (Fig 1A), which agreed with the trends of changes of inbreeding coefficients in the Jersey population. Using principal components based on ROH, the correlation of PC1 and FPED was high (r = 0.68), whereas correlations of FPED and PC2-PC5 did not exceed 0.1. Based on SNP genotypes, sub groups were not clearly separated by two principal components PC1 and PC2 (Fig 1B).

### Association of local autozygosity and inbreeding coefficient

**Fig 2:**
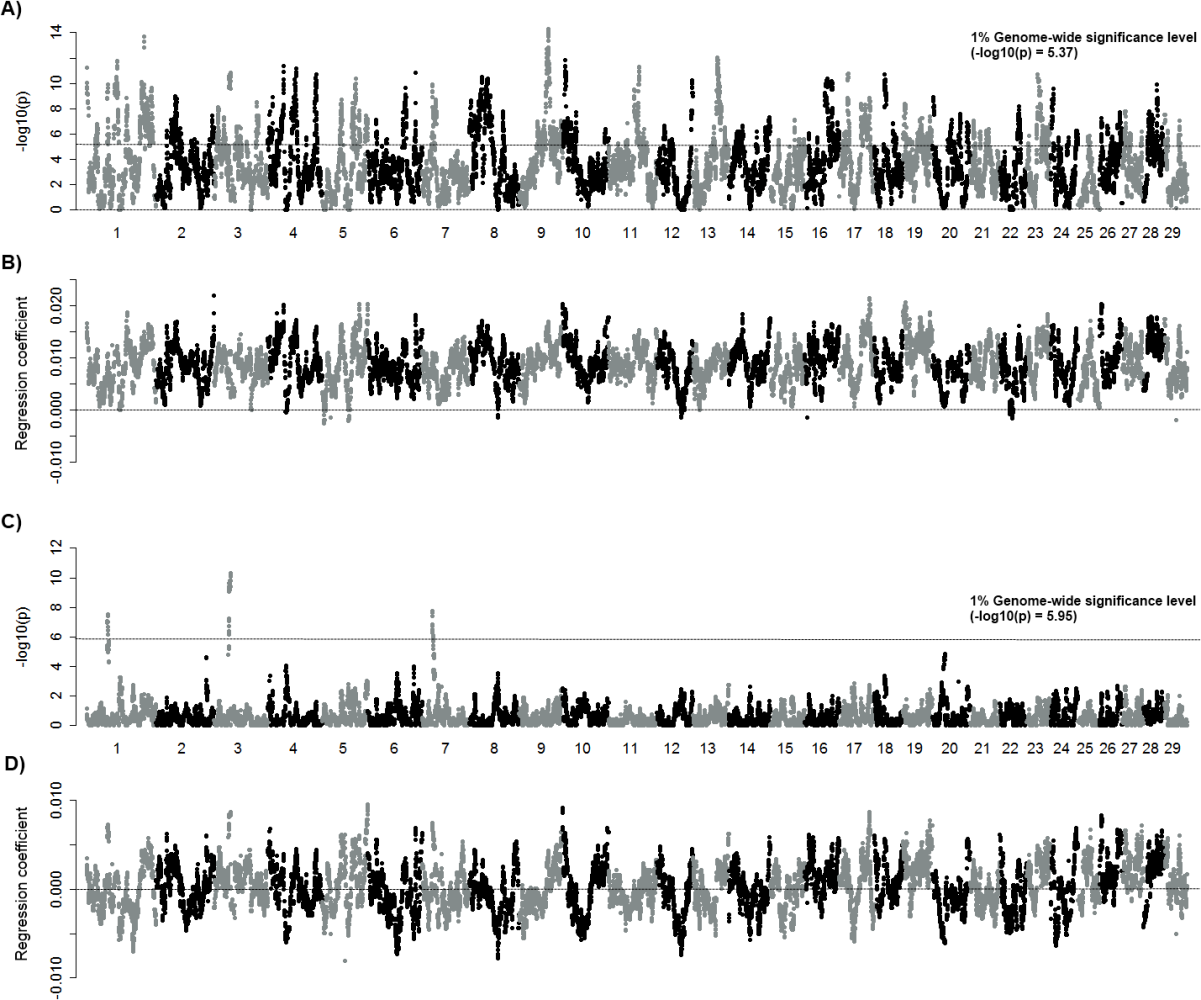
Genome-wide associations of ROH and *F*. Associations of ROH-FPED (-log_10_p) are plotted against each SNP locus across the genome (A). The y axis (B) represent the effect (slope) of ROH-FPED association and a dotted line indicates the effect = 0 (B). Genome-wide suggestive level is–log_10_p = 4.3, and the significant threshold (–log_10_p = 5.4) is shown with dotted line (A). PCA based adjusted associations between ROH and FPED (C) and its effect (D) are plotted. A dotted line displays genome-wide significance level (C).

The existence of substantial amounts of variation in the levels of ROH leads us to examine that particular genomic regions were more likely to contribute to changes of the inbreeding coefficient. Associations of ROH and pedigree-based inbreeding coefficients (ROH-FPED associations) were assessed to identify genomic regions accounting for FPED increases. This analysis revealed ROH levels had increased at 60 or more regions (≥1 Mb, genome-wide threshold *p*≥0.01) with increasing FPED during the last five decades (Fig 2A; Fig 2B; S1 Table). Despite weak statistical evidence, the levels of ROH in most regions (>99%) have increased concomitant with inbreeding coefficient (FPED), whereas ROH of a few genomic regions decreased as inbreeding increased but with weak statistical significance (Fig 2B). Notably, autozygosity in the major histocompatibility complex region (MHC, 25–30 Mb) on BTA 23 has increased as levels of inbreeding elevation, which may affect immune response to infectious disease like somatic cell score (SCS) that is an indicative parameter of mastitis in cattle.

In contrast, ROH-FPED association test carried out with five principal components (PC1-PC5), which account >90% of the total variation, revealed only a few regions that were significantly associated with FPED (Fig 2C). Three regions with high levels of ROH were on BTA 1 (49–50 Mb), BTA 3 (39–44 Mb), and BTA 7 (24–26 Mb) and were significantly associated with FPED regardless of models, whereas most associations were influenced by potential stratification. These regions were concordant with the high levels of ROH (>0.4), but not all regions with high levels of ROH agreed with ROH-FPED association. Interestingly, half of the regions of increased ROH were due to decreased levels of FPED when stratification was adjusted (Fig 2D), demonstrating the existence of principal components that are involved in the associations of ROH and inbreeding. Using the same analysis, the effects of PCs based on genotypes were not considerable.

### Mapping of ROH and DPR or SCS

**Fig 3:**
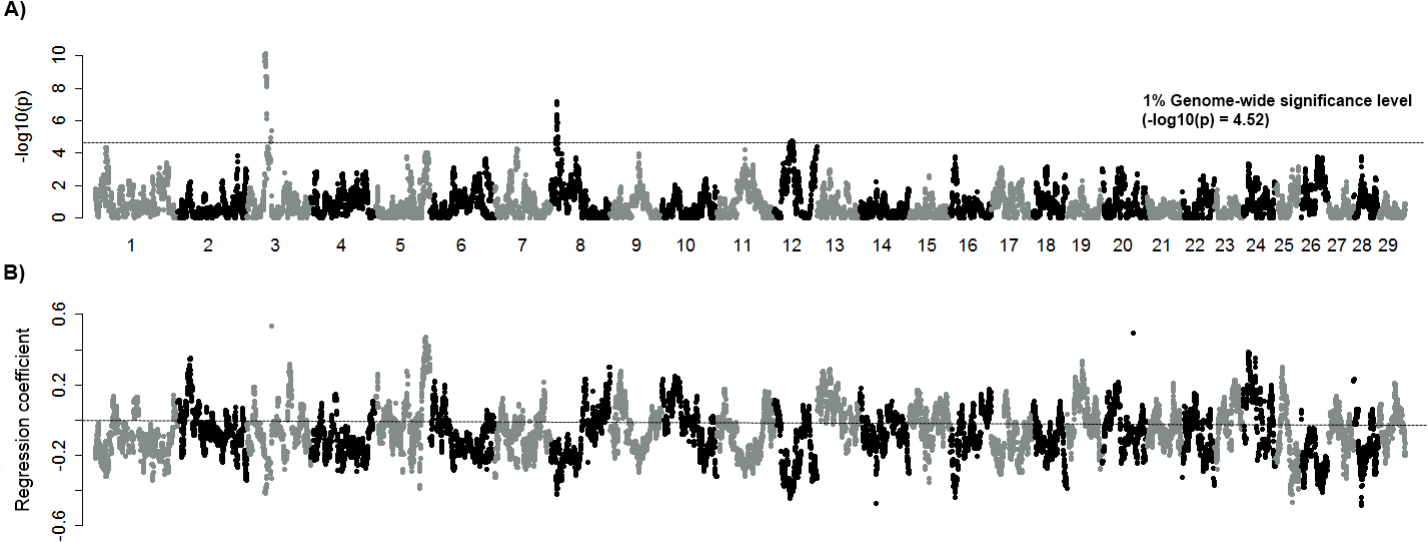
ROH-DPR associations. A) Significance of ROH-DPR association B) Effect of ROH-DPR association. A dotted line shows genome-wide significant threshold (adjusted 1% level, A). The positive or negative effect of daughter pregnancy rate (DPR) and ROH, which are defined by the slope of regression is plotted across the genome (B). Negative effect represents the region with decreased the levels of DPR by increased levels of ROH.

Next, the association of the ROH and traits, including DPR (ROH-DPR associations) or SCS (ROH-SCS associations) were examined across the genome using linear regression, and the results identified genomic regions affecting DPR (Fig 3A). ROH was associated with both increased and decreased DPR across the genome (Fig 3B). The considerable negative associations between ROH and DPR were found on BTA 3, 7, 8, and 12 (Table 1). In these regions, DPR has decreased as the level of ROH has significantly increased with pedigree based inbreeding coefficient (FPED), suggesting a potential influence of local autozygosity on fertility. Similarly, association test of somatic cell score (SCS) resulted in the directional effect of ROH on the trait (S2 Fig). ROH that were associated with SCS also affected increased FPED on BTA 1, 3, 4, 5, 13, and 21 (Table 1), suggesting that elevated homozygosity could be involved in the susceptibility to mastitis. A region at 40 Mb overlapped with a relatively broad region (>3 Mb) encompassing ROH-DPR associations on BTA 3. In this region (39–44 Mb) of BTA 3, ROH elevated also with increasing inbreeding coefficient (Table 1). *VCAM1* and *SLC35A3* are closely located between 42 and 43 Mb on BTA 3. There was an overlap between ROH-DPR and ROH-SCS associations at a region on BTA 7 but ROH was not associated with FPED in this region.

**Table 1:** Genome-wide association between ROH and DPR or SCS.

Trait^1^	BTA	Position (Mb)^2^	-log_10_(p)^3^	Effect	Position (Mb)	*F*_*L*_ ^4^	ROH-FPED^5^
**DPR**	3	40.04–44.47	10.06	-0.41	40.89	0.55	10.53**
		53.36–54.68	5.41	-0.33	54.65	0.27	1.27
	7	42.54–45.14	4.28	-0.27	44.53	0.61	1.02
	8	10.53–16.60	7.14	-0.42	13.81	0.20	7.01**
	12	34.66–42.08	4.78	-0.40	40.37	0.13	5.26**
		87.75–89.21	4.39	-0.33	89.09	0.20	9.27**
**SCS**	1	50.73–53.53	4.99	0.04	52.68	0.46	6.01**
		132.26–135.40	5.17	0.05	134.65	0.27	8.93**
	3	40.04–44.07	6.41	0.05	43.16	0.54	9.82**
	4	8.07–12.51	9.04	0.07	8.07	0.26	6.43**
	5	81.71–82.58	4.61	0.05	81.71	0.21	3.03**
	7	40.27–45.14	4.65	0.04	41.19	0.61	0.96
	13	58.05–59.22	5.32	-0.06	58.72	0.16	4.47*
	21	11.08–12.75	5.42	0.06	11.08	0.16	6.82**
		25.03–25.94	4.58	0.06	25.04	0.12	5.54**

^1^DPR = daughter pregnancy rate; SCS = somatic cell score.

^2^Regions was defined by genome-wide suggestive level of ROH-trait associations.

^3^Maximum association in the region.

^4^ *F*_*L*_^=^ ROH of a SNP locus.

^5^**1%, *5%, genome-wide thresholds of association between ROH and FPED without considering stratification. The value shows-log_10_(p-value).

The genes identified in the regions that were significantly associated with DPR or SCS and the respective biological pathways are listed in S3 and S4 Tables. The total size of the regions was less than 20 Mb, which is approximately 2% of the total size of genome, encompassing approximately 100 annotated coding regions. One of the largest clusters from the analysis of DPR and SCS, genes affecting cell communication and sensory cognition (*COL4A2*, *COL4A1*, *GJA2*, *GJB3*, and *GJB6*) are located on BTA 3 (~40 Mb). Annotation of the regions that were associated with SCS revealed several interesting genes that may be involved in biological pathways like immune response, including *CBLB* (BTA 1) and *NCK1* (BTA 1) that are involved in T cell receptor signaling pathway.

### Comparisons of ROH associated with phenotypes, F, and birth year

**Fig 4:**
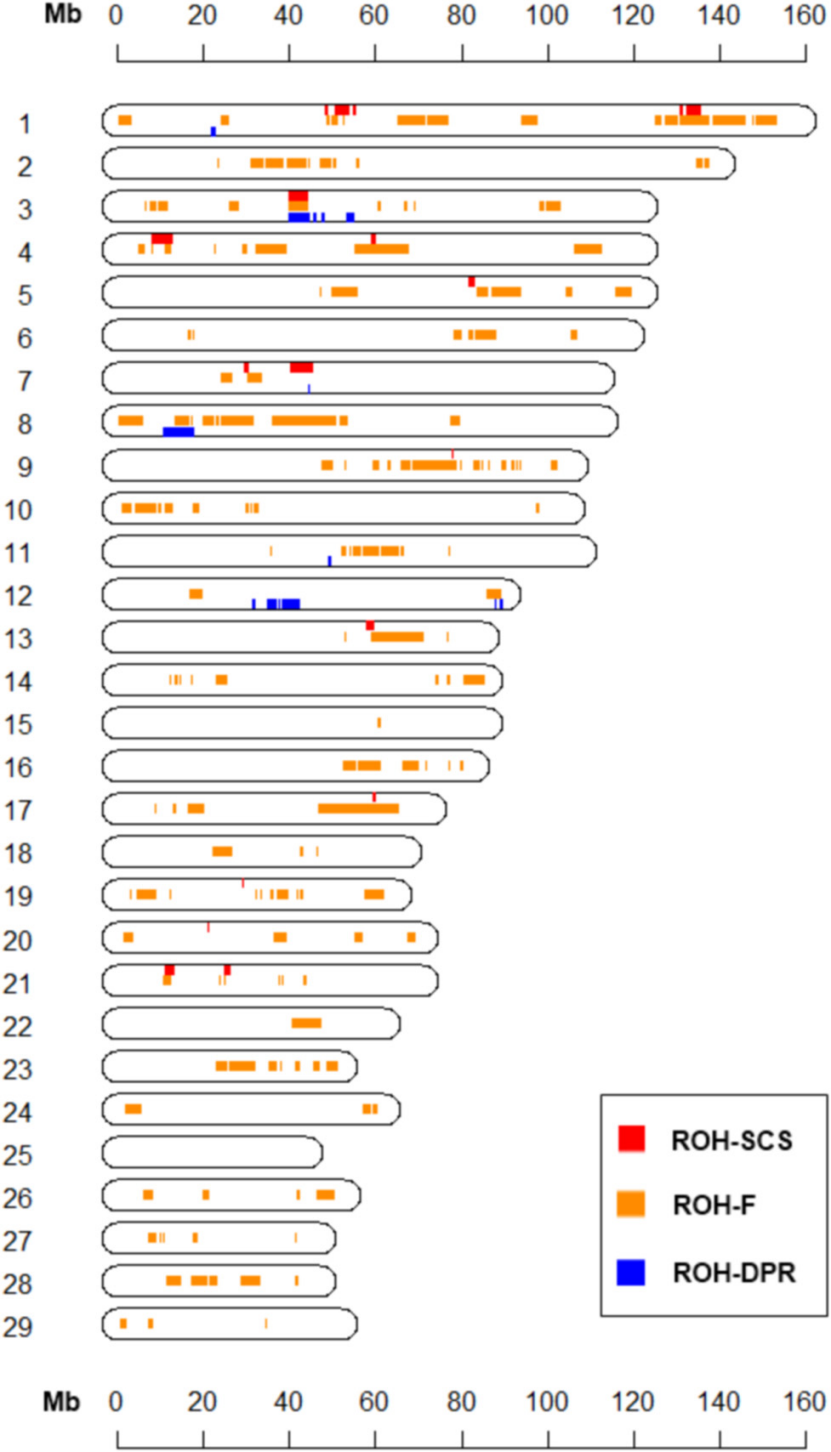
Comparisons of ROH-DPR, ROH-SCS and ROH-FPED. On each chromosome, red (upper), orange (middle) and blue (lower) bar display the significant associations of ROH-SCS, ROH-FPED, and ROH-DPR, respectively. The chromosome number is shown on the left side of each chromosome. Horizontal scale on top and bottom indicates genomic position (Mb) on a chromosome.

When comparing the regions significantly associated with traits or *F*, ROH that affected the change of inbreeding coefficient did not greatly influence the traits known to be influenced by inbreeding depression in most genomic regions (Fig 4). However, the association of ROH and DPR appeared to be related to local autozygosity that has increased with overall inbreeding at six regions. To assess the correlation further, a genome-wide correlation coefficient was calculated between the associations. Overall, moderate correlations were found between the results from genome-wide associations of ROH and traits, birth year, and inbreeding coefficient using regression coefficients (S2 Table). The effects of ROH-FPED associations and ROH-DPR associations were correlated negatively (-0.24). The association between birth year and ROH, which reflects the consistent change of ROH during the past few decades, was correlated with the effect of associations of ROH-SCS (0.43) or ROH-DPR (-0.28), respectively.

Finally, it is noted that ROH-FPED correlated with associations of ROH and the birth year of animals positively (0.36). This analysis examines the considerable change of ROH during the last five decades, which represents the regions under directional selection. Inbreeding coefficients in dairy cattle have increased mostly by selection of phenotypically superior ancestors that were frequently used as parents the next generation. When considering the annual change of ROH across the genome, several local autozygosity appeared to be involved in inbreeding and the increased level of ROH on BTA 2, 3, 5, 7, 8, 9, 16 and other regions (Table 2, S3 Fig), which implies that local autozygosity may be dependent on other genetic forces such as selection. On the other hand, FPED has been substantially increased with the elevating levels of ROH on several chromosomal regions, including BTA 4, 9, 10, 11, 13, 17, and 28, whereas the levels of ROH have not significantly associated with birth year in the same regions since the 1960s (S3 Fig; Fig 4).

**Table 2:** ROH associated with FPED and birth year of animals.

BTA	Position (>1Mb)^1^	Association^2^	Position (Mb)^3^	*F*_*L*_ ^4^
2	128.3–129.8	5.83	128.92	0.32
3	39.3–44.1	10.71	42.22	0.55
5	85.4–85.9	8.44	85.41	0.15
7	30.1–33.5	8.47	32.71	0.30
8	26.7–31.1	6.47	27.77	0.12
8	44.8–50.2	7.09	49.91	0.16
9	60.5–72.2	7.94	66.88	0.15
16	52.2–61.5	9.89	58.43	0.15
17	16.6–19.4	7.49	17.03	0.18
18	23.2–26.4	8.65	25.94	0.19
23	25.9–27.0	10.36	26.40	0.14
24	57.9–61.5	6.27	60.14	0.13

^1^ Region is defined by the common regions of genome-wide ROH-FPED associations and significant associations of birth year and ROH.

^2^ Genome-wide associations of ROH and FPED **(-log**_**10**_ **p)**.

^3^ Effect (slope) and position of the most significant association of ROH and FPED.

^4^ *F*_*L*_ = Level of ROH at the locus with the most significant association.

### Effect of haplotypes affecting variation in fertility

To examine the genetic effect mode of ROH, the correlation of the most frequent haplotype and ROH was assessed using haplotype homozygosity (HH) and then an association test between the most frequent haplotype and a trait was conducted. The HH calculated with a sliding window approach was then compared with ROH based homozygosity, allowing us further understanding of ROH. With a 50-SNP sliding window, mean length of haplotype was 3.4 Mb (standard deviation = 0.52) with an average of 123.5 (standard deviation = 77.0) alleles per haplotype (Table 3). The correlation among all ROH and HH was high (r = 0.9). In addition, no obvious large outlier was observed when comparing ROH and HH in Jersey cattle (S4 Fig). Although the average number of alleles was high, distribution of haplotype frequency was typically decided by a few crucial alleles. The mean frequency of the most frequent allele was 0.24 across the genome, having a range of 0.03 to 0.81. When all of the observed HH of each allele was calculated, the 5 most frequent alleles accounted for 90% of observed HH of all alleles (Table 3). For each haplotypic allele, observed HH did not deviate greatly from expected HH except the most frequent haplotype.

**Table 3:** Summary of the most frequent haplotypic allele using 50 SNP window.

Allele^1^	Allele 1	Allele 2	Allele 3	Allele 4	Allele 5	Sum
Mean frequency^2^	0.215 (±0.10)	0.126 (±0.04)	0.091 (±0.03)	0.070 (±0.02)	0.054 (±0.02)	0.556
Maximum frequency	0.78	0.35	0.18	0.15	0.10	-
Homozygosity (Exp)^3^	0.046	0.016	0.008	0.005	0.003	0.080
Homozygosity (Obs)^4^	0.053	0.018	0.008	0.005	0.003	0.087
Homozygosity (Obs%)^5^	54.2	18.4	8.2	5.5	3.2	89.5

^1^ Allele 1 to 5 was assigned by the descending order of haplotype frequency.

^2^ Mean of expected frequency/homozygosity and standard deviation without inbreeding.

^3^ Mean of expected homozygosity.

^4^ Mean of observed homozygosity.

^5^ Percentage of observed homozygosity in homozygosity of all haplotypes.

**Fig 5:**
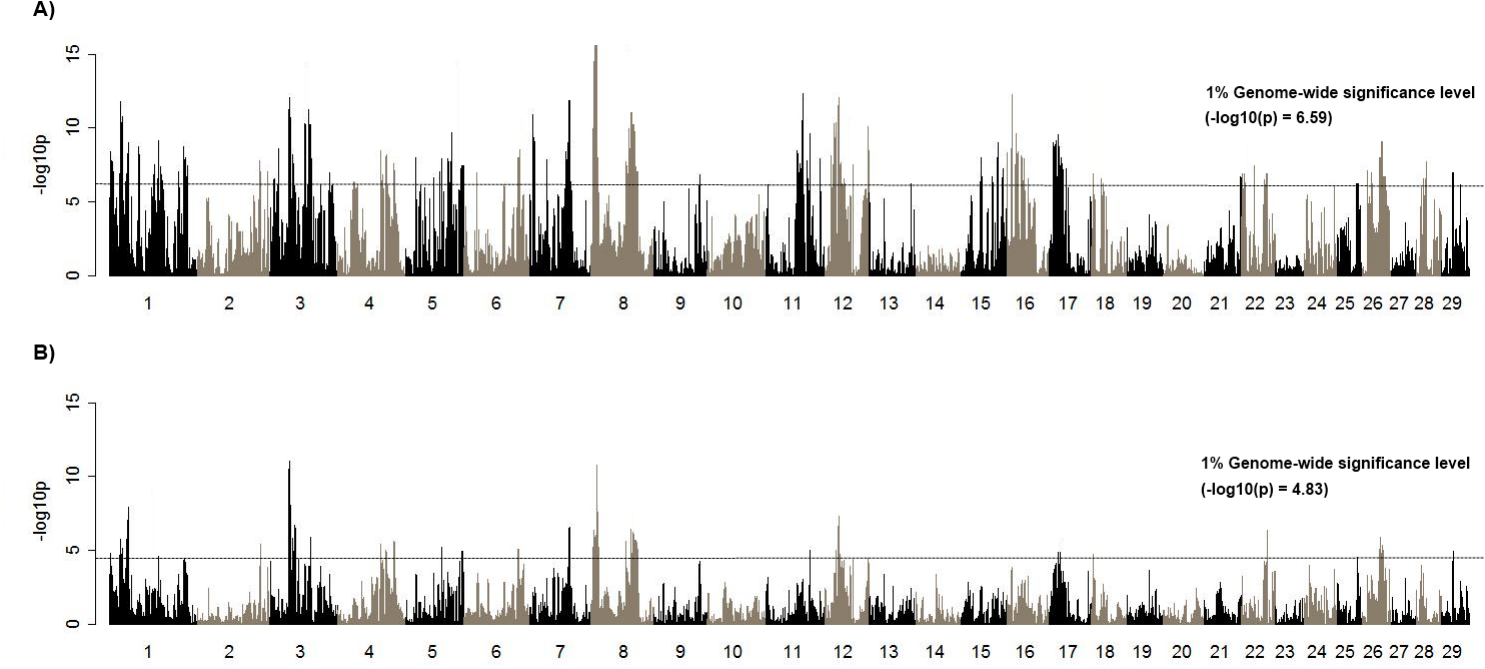
Association between DPR and the most frequent haplotype using additive (A) and recessive model (B). Each bar demonstrates the association of DPR and haplotype that is defined by the 50-SNP window. Association of DPR and the most frequent haplotype (A) or homozygous status of the most frequent haplotype (B) represents an additive or recessive effect. Genome-wide significance level is shown on each plot.

When testing the association between ROH and fertility, we found that many regions with high or moderate levels of ROH were associated with fertility positively, suggesting that the genetic effect of most ROH appears to be unrelated to decreasing DPR. In Fig 5A, the significance for the regression showing the additive effect of the most frequent effect on DPR is shown, and in Fig 5B the significance of HH is seen. It was expected that results of the recessive model largely overlapped with regions harboring significant associations of ROH-DPR and correlation of associations from two models was considerably high (r = 0.8). Additionally, even the results from additive genetic model agreed for the regions that were associated with DPR using the recessive genetic model, in addition to the other region influencing fertility additively. In all, our results suggest that associations between ROH and DPR are concordant with the results from either the additive or recessive effects model using the most frequent haplotype, implying that ROH-DPR associations could be interpreted as quantitative trait loci as well as evidence of inbreeding depression due to increased homozygosity.

## Discussion

A deleterious mutation may emerge randomly or one that exists may increase in frequency due to close linkage with a favorable allele under strong selection [30]. Because thousands of genes are influenced by the opposite effects of mutation and selection, detrimental or lethal alleles could be quite important when considering the entire genome [4]. In a highly redundant system, a deleterious mutation in a single gene may have a negligible effect and selection can only operate against the combination of mutations [3]. In contrast to random mating, artificial selection has increased overall similarity of the whole genome in livestock, particularly the genomic regions influencing economic traits [31].

Some inbreeding depression has been shown to be due to rare large-effect viability and fertility mutations in dairy cattle [32, 33]. However, in general inbreeding depression appears to be contributed to by rare, mildly detrimental mutations at many loci [34]. The sum of the mean number of deleterious causative polymorphism in zygotes, which contribute to the next generation has been estimated to be 12–32 in the human genome [3]. Inbreeding depression is a universal phenomenon, whereas the influence of inbreeding depression varies for different species and even for different populations of the same species [35]. VanRaden and colleagues [36] identified five haplotypes carrying lethal alleles in each dairy cattle breed including Holstein, Jersey, and Brown Swiss in the United States. However, the number of moderate and incomplete recessive deleterious mutation has not clarified in cattle.

Specifically, more than 60 regions showed a considerable change of local autozygosity accompanied by an elevation of inbreeding during the last few decades in Jersey cattle. On the contrary, only a small amount of ROH was associated with lower fertility in contemporary Jersey cattle. In addition, regions with high ROH (>0.3) created by recent inbreeding have not always been detrimental. In Australian Jersey cattle, only one genomic region affecting fertility was detected on the X chromosome using ROH based test [37]. Systematic mating may reduce relatedness between family members [38], but genomic factors corresponding to inbreeding depression have not been adequately characterized. The inverse correlation between milk production and fertility and the general belief that reproductive traits are associated with inbreeding depression led us to hypothesize that reproduction in cattle is associated with regions with excessive or increased levels of ROH in dairy cattle. It is worth noting that the high frequency of long intact haplotypes carrying loci under selection is one of the best determinants of selection [6].

Inbreeding coefficient (FPED) tends to be affected by influential and recent common ancestors, which may include stratification. According to the PCA based on genotypes, there was no obvious structure in Jersey data. However, the association test including or excluding stratification suggested to us some debatable results. After removing stratification using PCA of ROH, only three regions appeared to be bound to change of inbreeding coefficient, which deviated from our expectation. Interestingly, the correlation of the first principal component (PC1) and FPED was 0.7, which suggests that PC1 may account for the large amount of variation due to inbreeding coefficient (FPED). Therefore, we focused on the results from the analyses without PCs.

The correlation between the genomic and pedigree inbreeding coefficient is less than 0.7 in Jersey cattle, which is lower than the value observed in small human families (~0.85) [10]. The correlation between FPED and genotype based inbreeding coefficients were 0.74 using true allele frequencies and 0.68 using estimates of base frequencies in dairy cattle [39]. In Jersey cattle, correlation of FPED and FROH was 0.65 using 10–100 SNP thresholds [37], which agrees with our findings. A pedigree simulation study suggested that the variation in true autozygosity is considerably high among individuals with the same level of inbreeding without additional genetic effects like selection or migration [18]. Additionally, a pedigree based calculation of the inbreeding coefficient assumed founder animals were unrelated, which possibly results in lower *F* value compared to overall genomic autozygosity. In Jersey cattle, multiple regions showed substantial change of autozygosity during the last few decades, which suggested genetic forces like selection are more likely to play an important role in determining genome-wide patterns of autozygosity as well as inbreeding.

The local effect hypothesis assumes that heterozygosity-fitness correlations are the results of heterozygosity of particular genes and loci [40]. A mutation in *SLC35A3* on BTA 3 causes complex vertebral malformation (CVM) [41], which is a recessively inherited disorder with the onset occurring during fetal development, and leading to frequent abortion of fetuses or perinatal death, and vertebral anomalies [1]. A causative mutation of CVM originated from only two influential sires born during the 1960s in the US. Similarly, alleles contributing to inbreeding depression in the US Jersey population appear to be associated with particular specific founders [41]. Another spontaneous mutation may occur in *SLC35A3* or a neighboring gene such as vascular cell adhesion molecule-1 (*VCAM1*) that mediates leukocyte recruitment from blood into tissues and affects embryo survival rate in the mouse [42]. Decreases in lethal alleles from selection or inbreeding may lead to an apparent deficiency of heterozygotes compared to Hardy-Weinberg expectations [43]. Lethal genes have been reported in >5,000 Jersey cattle [36, 44], but minor alleles at a low frequency (< 0.1) are rarely detected using the HWE test in a small number of animals. In the case of the pedigree structure in domestic animals, multiple alleles appear to be common at major gene loci under selection for many generations [45]. This may restrict the increasing rate of a recessive mutation near favorable loci unless the mutation exists in all haplotypes carrying the loci under selection.

Our primary aim was to identify the regions corresponding to local autozygosity by detecting associations of ROH and DPR or SCS. Despite the fact that generally the inbreeding coefficient is the most influential factor associated with the decline in fertility, reproduction traits are generally quantitative traits and are largely affected by environment [46]. QTL mapping has been used to identify the genetic mode inducing inbreeding depression in *Drosophila* [47], demonstrating incomplete action of recessive effects on viability. The effect of ROH was expected to be partially or nearly recessive thus contributing to inbreeding depression. As inbreeding depression is also a consequence of nonlinear interactions between gene effects, it stands to reason that epistasis may complicate the analysis and interpretation [48]. Despite some ROH decreasing as FPED increased, most ROHs were positively correlated to FPED, supporting partial dominance rather than an overdominance hypothesis [14] Using haplotypes, the additive effects model explained variation in fertility better compared to the recessive effects model. Alternatively, the genetic effects estimated using a recessive model may be estimated approximately through the use of an additive model. For more accurate estimation, daughter yield deviation (DYD) of DPR that accounts for the additive effect and inbreeding depression can be used instead of PTA, but most of the recent genetic trend is decided by the additive effect [16]. It is important to recognize that most genetic effects will be additive when the mutations are incompletely recessive [48]. The power to detect recessive causal alleles is poor when the minor allele frequency was not close to 50% [49]. Estimated non-additive genetic variances for fertility were not smaller than the additive genetic variance in Holstein cattle [50]. Thus, an optimum approach will be necessary to evaluate precisely the effect of local autozygosity on traits.

In humans, susceptibility of disease increased with the increase of overall autozygosity, but a local effect of autozygosity on a disease has not been clarified [26]. In the primary analysis, we regarded the region at a high or increasing frequency of ROH as a product of an elevated inbreeding coefficient. However, the frequencies of alleles do not change significantly without other genetic events, whereas genotypic frequencies change due to inbreeding, which leads us to consider an alternative explanation of associations of ROH and FPED. In addition, the amount of change in local autozygosity does not necessarily agree with increasing levels of inbreeding. This suggests that inbreeding does not solely drive an increase of a local ROH faster than the other regions. Thus, we attribute locally emerging ROH to other genetic events including recent selection and inheritance from the recent common ancestors. The comparison between signatures of selection in Holstein cattle and ROH-FPED associations showed consistency on several regions which accounted for variation in milk yield [13]. The correlation of ROH-FPED associations and ROH-birth year associations (0.32–0.36) was considerable, which appears to be bound to an increase in the most frequent haplotype. This may suggest that multiple genetic events such as artificial selection based on a small number of effective founder animals, resulting in changing levels of ROH.

If records are not available, ROH appears to be the most powerful method for analysis of detecting inbreeding effects from among several alternative estimates of *F* with large sample size (n>12,000) [22]. However, contrary to expectations, association mapping of ROH with traits has demonstrated several additive QTL effects of the most frequent haplotypes, in particular when ROH was positively associated with DPR. The QTL accounting for fertility and calving traits have been reported in dairy cattle [46, 51]. In the US Holsteins, the top 10% of bulls had a DPR 4.9% higher than bulls in the lowest 10% [31], which could be exploited to improve reproduction and which may explain why not all regions of ROH were negatively associated with fertility problems.

We have suggested methods for assessing the relationships between inbreeding, genomic autozygosity, and trait performance using prior information from a Jersey cattle population. ROH could be considered the optimal one to measure inbreeding based on genomic information [11]. A survey of runs of homozygosity using high density SNP provided insight into the formation and role of autozygosity. However, the reasons for variation of the distribution and formation of ROH are unclear [14]. ROH appears to originate from selected ancestors that are likely to be related to each other, which suggests that the association between ROH and inbreeding coefficient appears to be indicative of the evidence of recent selection resulting in increased inbreeding. Altogether, we conclude that ROH reflects the homozygosity largely influenced by recent artificial selection [52, 53], suggesting the need for a careful interpretation of results from the analysis of associations between ROH and traits that is related to inbreeding depression in the US Jersey cattle.

## Supporting Information

S1 Table Correlation and regression of FROH on FPED. ^1^Definition of ROH based on the number of continuous homozygous SNP (30, 40, and 50 SNPs) or size (3 or 5 Mb). ^2^Regression coefficients of FPED on FROH are shown.
(DOCX)

S2 Table Correlation of associations between ROH and FPED, birth year, DPR, and SCS. Correlation coefficient (r) are shown: r of the ROH-FPED, ROH-Year, ROH-DPR, and ROH-SCS represents associations between ROH and FPED, birth year, DPR, and SCS, respectively.
(DOCX)

S3 Table Genes in the regions encompassing associations between ROH and DPR. The function of gene is annotated using Enrichr. Genes annotated by KEGG database (www.kegg.org) are summarized in Excel file.
(XLSX)

S4 Table Genes in the regions encompassing associations between ROH and SCS. The function of gene is annotated using Enrichr. Genes annotated by KEGG database (www.kegg.org) are summarized in Excel file.
(XLSX)

S1 Fig Correlation of genomic autozygosity (ROH) and inbreeding coefficient. The y axis represents the pedigree inbreeding coefficient (FPED), and the y axis indicates the levels of genomic inbreeding based on ROH (FPED).
(TIF)

S2 Fig Associations of ROH and SCS. A) Significant level of associations, B) Regression coefficient of associations. A dotted line shows genome-wide significant threshold (adjusted 1% level, A). The positive or negative effect of daughter pregnancy rate (SCS) and ROH, which are defined by the slope of regression is plotted across the genome (B). Negative effect represents the region with decreased the levels of SCS by increased levels of ROH.
(TIF)

S3 Fig Genome-wide association of ROH and FPED or birth year. ROH-F associations are plotted with gray bars and associations of ROH and birth year are shown in black dots. The dotted line shows the genome-wide significance level of ROH-FPED associations.
(TIF)

S4 Fig The correlation between ROH and haplotype homozygosity. ROH (y axis) corresponding to sliding window is plotted against haplotype homozygosity (HH, x axis). Mean ROH of in a sliding window is calculated to compare with 50-SNP haplotype homozygosity.
(TIF)

S1 File Data availability. (DOCX)
