# Supplementary material for: The Relationship between Runs of Homozygosity and Inbreeding in Jersey Cattle under Selection
Source: PLoS One. 2015 Jul 8;10(7):e0129967. doi: 10.1371/journal.pone.0129967 (PMC4496098; doi:10.1371/journal.pone.0129967)
Supplement: S1 Table — 1Definition of ROH based on the number of continuous homozygous SNP (30, 40, and 50 SNPs) or size (3 or 5 Mb). 2Regression coefficients of FPED on FROH are shown. (DOCX) [file pone.0129967.s001.docx]

**S1 Table. Correlation and regression of FROH on FPED.**

| **Thresholds for ROH^1^** | **30 SNPs** | **50 SNPs** | **80 SNPs** | **100 SNPs** |
| --- | --- | --- | --- | --- |
| Correlation (r) | 0.71 | 0.71 | 0.71 | 0.70 |
| Slope^2^ | 0.90 | 0.90 | 0.87 | 0.85 |
| Intercept^2^ | 0.12 | 0.09 | 0.06 | 0.05 |
| Mean size of homozygous segments (Mb) | 6.23 | 8.48 | 11.60 | 13.57 |
| Median size of homozygous segments (Mb) | 4.16 | 6.09 | 8.93 | 10.68 |
| Minimum size of homozygous segments (Mb) | 0.98 | 1.85 | 2.93 | 3.78 |
| Mean levels of ROH | 0.17 | 0.14 | 0.12 | 0.10 |

^1^Definition of ROH based on the number of continuous homozygous SNP (30, 40, and 50 SNPs) or size (3 or 5 Mb). ^2^Regression coefficients of FPED on FROH are shown*.*
