# Supplementary material for: The Relationship between Runs of Homozygosity and Inbreeding in Jersey Cattle under Selection
Source: PLoS One. 2015 Jul 8;10(7):e0129967. doi: 10.1371/journal.pone.0129967 (PMC4496098; doi:10.1371/journal.pone.0129967)
Supplement: S2 Table — Correlation coefficient (r) are shown: r of the ROH-FPED, ROH-Year, ROH-DPR, and ROH-SCS represents associations between ROH and FPED, birth year, DPR, and SCS, respectively. (DOCX) [file pone.0129967.s002.docx]

**S2 Table. Correlation of associations between ROH and FPED, birth year, DPR, and SCS**

| **Association** | **ROH-FPED** | **ROH-Year** | **ROH-DPR** |
| --- | --- | --- | --- |
| **ROH-Year** | 0.36 |  |  |
| **ROH-DPR** | -0.24 | -0.28 |  |
| **ROH-SCS** | 0.08 | 0.43 | -0.25 |

Correlation coefficient (r) are shown: r of the ROH-FPED, ROH-Year, ROH-DPR, and ROH-SCS represents associations between ROH and FPED, birth year, DPR, and SCS, respectively.
