# Supplementary figures and images for: The Relationship between Runs of Homozygosity and Inbreeding in Jersey Cattle under Selection
Source: PLoS One. 2015 Jul 8;10(7):e0129967. doi: 10.1371/journal.pone.0129967 (PMC4496098; doi:10.1371/journal.pone.0129967)

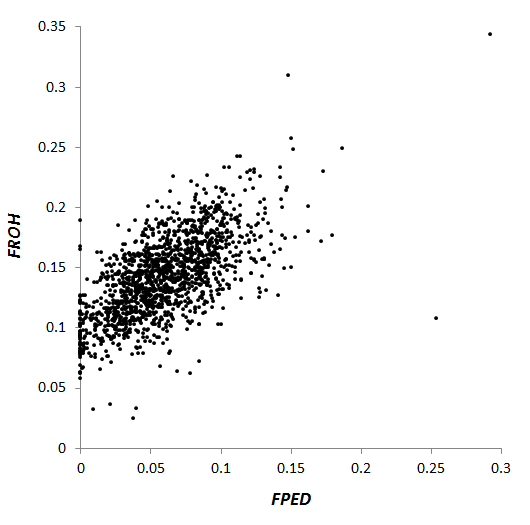

Supplement: S1 Fig — The y axis represents the pedigree inbreeding coefficient (FPED), and the y axis indicates the levels of genomic inbreeding based on ROH (FPED). (TIF) [file pone.0129967.s005.tif]

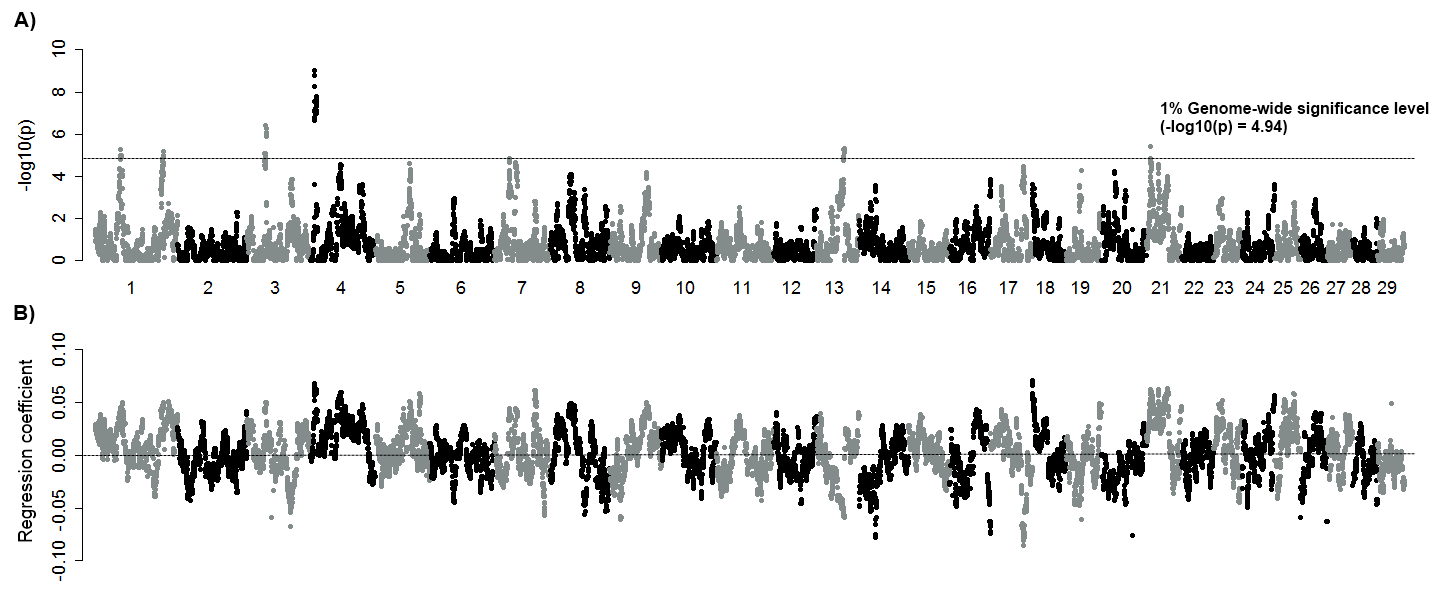

Supplement: S2 Fig — A) Significant level of associations, B) Regression coefficient of associations. A dotted line shows genome-wide significant threshold (adjusted 1% level, A). The positive or negative effect of daughter pregnancy rate (SCS) and ROH, which are defined by the slope of regression is plotted across the genome (B). Negative effect represents the region with decreased the levels of SCS by increased levels of ROH. (TIF) [file pone.0129967.s006.tif]

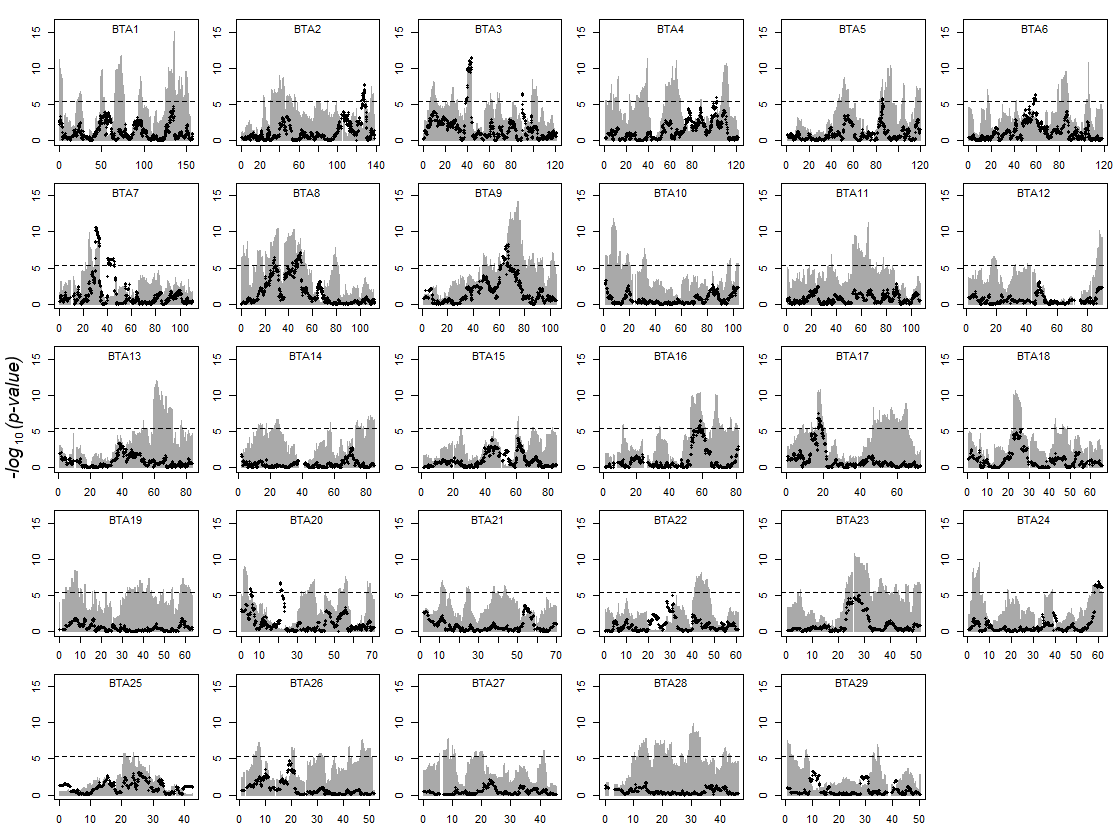

Supplement: S3 Fig — ROH-F associations are plotted with gray bars and associations of ROH and birth year are shown in black dots. The dotted line shows the genome-wide significance level of ROH-FPED associations. (TIF) [file pone.0129967.s007.tif]

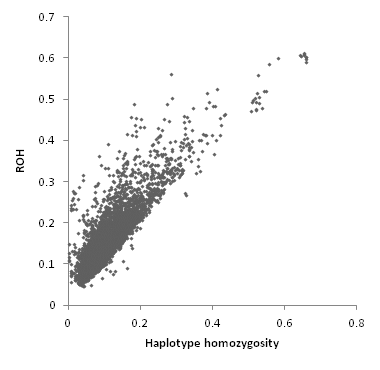

Supplement: S4 Fig — ROH (y axis) corresponding to sliding window is plotted against haplotype homozygosity (HH, x axis). Mean ROH of in a sliding window is calculated to compare with 50-SNP haplotype homozygosity. (TIF) [file pone.0129967.s008.tif]
