# Supplementary material for: The Relationship between Runs of Homozygosity and Inbreeding in Jersey Cattle under Selection
Source: PLoS One. 2015 Jul 8;10(7):e0129967. doi: 10.1371/journal.pone.0129967 (PMC4496098; doi:10.1371/journal.pone.0129967)
Supplement: S1 File — (DOCX) [file pone.0129967.s009.docx]

**Plos One Revision Data Availability**

**The data used in this study were generated in a collaboration between USDA-ARS scientists and the National Association of Animal Breeders (NAAB) under a Cooperative Research and Development Agreement (CRADA) that has since expired. This CRADA granted governance of this data to NAAB. As part of the CRADA, USDA-ARS scientists could access this genomic data for the purpose of genomics research and genomic evaluations of US Jersey cattle.**The recently formed Council on Dairy Cattle Breeding LLC (CDCB) manages the dairy industry’s genotypic and performance data. The NAAB is a member organization of CDCB. Research access to the genotype data used in this study is controlled by the CDCB. The CDCB accepts research proposals that can include access to genotypic data.
